# The complete mitochondrial genome of the freshwater fish *Opsariichthys uncirostris amurensis* from Korea

**DOI:** 10.1080/23802359.2021.1959433

**Published:** 2022-02-03

**Authors:** Kang-Rae Kim, Yong Hwi Kim, Sung Mu-Sung, Jong Yeon Park, In-Chul Bang

**Affiliations:** Department of Life Science and Biotechnology, Soonchunhyang University, Asan, Republic of Korea

**Keywords:** Complete mitochondrial genome, Cyprinidae, *Opsariichthys uncirostris amurensis*, freshwater fish

## Abstract

We present the first report of the complete mitochondrial genome of *Opsariichthys uncirostris amurensis*, which consists of 16,613 base pairs harboring 13 protein-coding genes, two ribosomal RNA genes, 22 transfer RNA genes, and a control region (D-loop). The overall base composition of the complete genome is A (27.15%), C (27.15%), T (26.77%), G (18.92%). The complete mitogenome of *O. uncirostris amurensis*, which was most closely related to *O. bidens* in the Bayesian inference tree, provides a better understanding of the phylogeny of the genus *Opsariichthys*.

*Opsariichthys uncirostris amurensis* (Berg 1932) (Cypriniformes: Cyprinidae) is a subspecies of *O. uncirostris*. *Opsariichthys uncirostris amurensis* lives in most rivers and streams in the Korean Peninsula, except for those flowing to the East Sea of Korea (Kim and Park 2002). *Opsariichthys uncirostris amurensis* has been studied only in phylogenetic analyses of the genus *Opsariichthys*; studies of *O. uncirostris amurensis* are lacking (Okazaki et al. 2002). This study is the first to report the complete *O. uncirostris amurensis* mitogenome and provides a better understanding of the phylogenetic relationships in the genus *Opsariichthys*.

A specimen of *O. uncirostris amurensis* was collected from Jicheon Stream (36°19'N, 126°55'E) flowing into the Geumgang River in Korea on 18 May 2018, and was preserved in 99.9% ethanol. The specimen was given voucher number SUC-25115 and stored in the fish specimen room of Soonchunhyang University, Korea (Voucher Storage: Soonchunhyang University; Voucher number: SUC-25115; The person in charge of the collection: KR Kim; email: kimkangrae9586@gmail.com). Genomic DNA was extracted from the caudal fin using the Genomic DNA Prep Kit (BioFact, Daejeon, South Korea) and stored in a deep freezer at −80 °C. Genomic DNA was extracted from the caudal fin using the Genomic DNA Prep Kit (BIOFACT, Daejeon, South Korea). The extracted genomic DNA was stored in the specimen room of Soonchunhyang University. To obtain the complete mitochondrial DNA sequence, a DNA library was prepared according to the MGIEasy DNA Library Prep Kit (MGI Tech, Shenzhen, China) manual and subjected to 150 bp paired-end sequencing on the MGISEQ-2000 platform (MGI Tech). The next-generation sequencing raw data were assembled using Geneious 11.0.3, and the assembled mitogenome sequences were annotated using the MITOS web server (Bernt et al. 2013). The complete mitogenome sequence of *O. uncirostris amurensis* was deposited in the National Center for Biotechnology Information GenBank database under the accession number MW355488.[Fig F0001]

The mitochondrial genome of *O. uncirostris amurensis* consists of 16,613 bp, harboring 13 protein-coding genes (PCGs), two ribosomal RNA (rRNA) genes, 22 transfer RNA (tRNA) genes, and a control region (D-loop). Of the 13 PCGs, only the CO1 gene started with GTG, while the remaining 12 started with ATG. Three PCGs (CO2, CO3, and Cytb) contain an incomplete stop codon (T), while 10 PCGs (CO1, ND1, ND2, ND3, ND4, ND4L, ND5, ND6, ATP8, and ATP6) contain a complete stop codon (TAA or TAG). The overall base composition of the *O. uncirostris amurensis* genome is A (27.15%), C (27.15%), T (26.77%), G (18.92%), with a high AT content of 53.92%. The two rRNAs are 16S (1,693 bp) and 12S (955 bp).

A phylogenetic tree was constructed based on the 13 PCGs using Bayesian inference in MrBayes 3.2.7 (Ronquist et al. 2012). For the dataset, we used the GTR + I+G model proposed in jModelTest 2.1.10 (Guindon and Gascuel 2003; Darriba et al. 2012).

**Figure 1. F0001:**
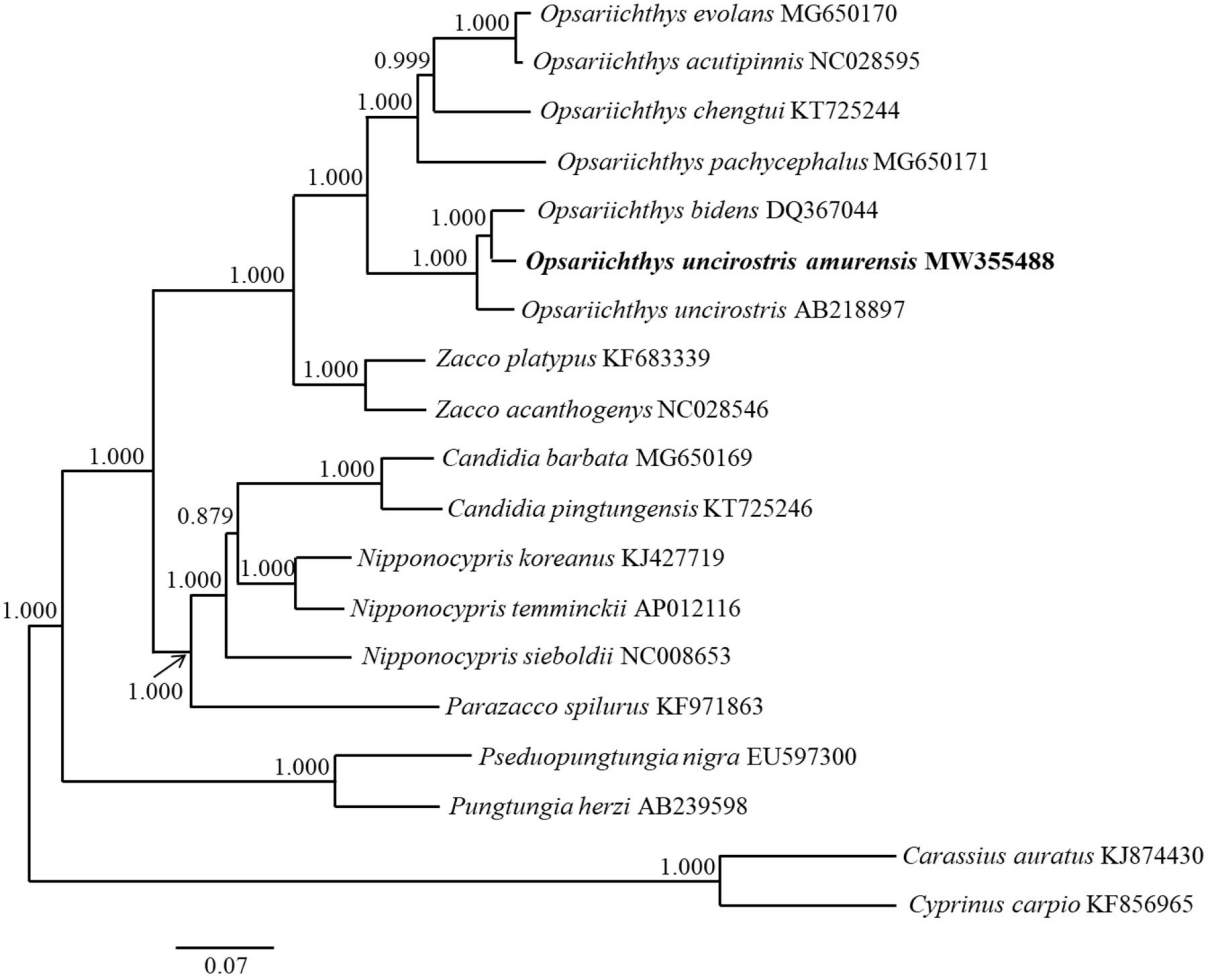
A phylogenetic tree was constructed for the genera *Opsariichthys, Zacco, Candidia, Nipponocypris, and Parazacco*, with outgroup species and subspecies, using Bayesian inference based on 13 protein-coding genes. The numbers above the nodes are the posterior probabilities of the Bayesian analysis. Scale bars indicate the relative evolutionary distances. The species names are followed by their GenBank accession numbers.

The results indicated that *O. uncirostris amurensis* is the most closely related to *Opsariichthys bidens* (Günther 1873). *Opsariichthys uncirostris amurensis* from the Korean Peninsula was expected to be close to *O. uncirostris*, which live in Japan, but was more closely related to *O. bidens* from China (Figure 1). When the sea level was low, the rivers flowing to the western part of the Korean Peninsula, eastern China, and western Japan were connected to each other through the paleo-Yellow River system. However, it is thought that the river flowing to western Japan was separated geographically due to the sea level rise, and the fish therein evolved into *O. uncirostris* (Okazaki et al. 2002; Takehana et al. 2004; Jung et al. 2009; Won et al. 2020).

For greater insight, a p-distance analysis was performed on *O. uncirostris amurensis* and species in the genus *Opsariichthys*. The p-distances between *O. uncirostris amurensis* and other species of the genus *Opsariichthys* ranged from 0.137 to 0.142, while *O. uncirostris amurensis* was 0.054 from *O. uncirostris* and 0.044 from *O bidens*. Interestingly, despite being a subspecies of *O. uncirostris*, *O. uncirostris amurensis* had a p-distance difference of the similar magnitude as *O. bidens*. Therefore, p-distance analysis of *O. uncirostris amurensis*, *O. uncirostris*, and *O. bidens* was performed using CO1 sequences in NCBI. The p-distance within *O. uncirostris amurensis* was in the range 0.002 ∼ 0.007 (HQ536417∼HQ536421, HQ536417∼HQ536421, and MK560566∼MK560568). The p-distance between *O. uncirostris amurensis* and *O. uncirostris* was 0.028 and 0.019 ∼ 0.031 for *O. bidens* (AB218897, LC098444, and MF122583∼MF122595). Therefore, *O. uncirostris amurensis* and *O. uncirostris* were recognized as subspecies, although the p-distance differs at the species level. Detailed morphological and molecular phylogenetic analyses are needed. The complete mitogenomes of *O. uncirostris amurensis*, *O. bidens*, and *O. uncirostris* will provide a better understanding of the phylogeny of this species and subspecies.

## Data Availability

The genome sequence data that support the findings of this study are openly available in GenBank of NCBI at (https://www.ncbi.nlm.nih.gov/) under the accession no. MW355488. The associated BioProject, SRA, and Bio-Sample numbers are PRJNA687317, SRX9720471 and SAMN17141024, respectively.
